# Efficacy and Safety of a Fixed-Dose Combination of Brinzolamide 1%/Timolol 0.5% vs. Dorzolamide 2%/Timolol 0.5% in Indian Patients With Primary Open-Angle Glaucoma or Ocular Hypertension: A Randomized Phase 3 Study

**DOI:** 10.7759/cureus.73599

**Published:** 2024-11-13

**Authors:** Purvi Bhagat, Mukesh Singhania, Sujata Navare, Mohua Mazumdar, Chandrima Paul, Satish Shet, Kalyani VKS, Anjali Nicholsan, Nitesh Bansal, Prachi Jain

**Affiliations:** 1 Ophthalmology, Civil Hospital Campus, Ahmedabad, IND; 2 Ophthalmology, Anand Multispeciality Hospital, Ahmedabad, IND; 3 Ophthalmology, Aster Aadhar Hospital, Kolhapur, IND; 4 Ophthalmology, Seth Sukhlal Karnani Memorial Hospital, Kolkata, IND; 5 Ophthalmology, B B Eye Foundation, Kolkata, IND; 6 Ophthalmology, Karnataka Institute of Medical Sciences, Hubli, IND; 7 Ophthalmology, Poona Blind Men's Association's H.V. Desai Eye Hospital, Pune, IND; 8 Ophthalmology, Topiwala National Medical College, Mumbai, IND; 9 Ophthalmology, Marudhar Hospital, Jaipur, IND; 10 Medical Affairs, Sun Pharma Laboratories Limited, Mumbai, IND

**Keywords:** brinzolamide-timolol fdc, dorzolamide-timolol fdc, intraocular pressure (iop), ocular hypertension, primary open-angle glaucoma (poag)

## Abstract

Introduction

Fixed-dose combinations (FDCs) have the potential in glaucoma management to improve efficacy due to the complementary mechanism of action of the drugs as well as compliance while reducing adverse effects by minimizing exposure to preservatives and the financial burden on the patients. FDC of brinzolamide/timolol has demonstrated efficacy and safety in multinational phase 3 studies in primary open-angle glaucoma (POAG) and ocular hypertension. However, efficacy and safety in the Indian population are not known. This study compared the efficacy and safety of FDC brinzolamide 1%/timolol 0.5% with FDC dorzolamide 2%/timolol 0.5% in Indian patients with POAG or ocular hypertension.

Material and methods

This 12-week randomized, phase 3, open-label, comparative, multicentric study was conducted on 221 subjects at nine sites in India, with assessments done at baseline and weeks 4, 8, and 12. Patients with intraocular pressure (IOP) of 24-36 mmHg of the affected eye(s), either newly diagnosed or inadequately controlled on mono-therapy of carbonic anhydrase inhibitor, beta-blocker, or any other IOP-lowering therapy, were included. Patients were randomly assigned to receive either FDC of brinzolamide 1%/timolol 0.5% (n = 111) or FDC of dorzolamide 2%/timolol 0.5% (n = 110). Primary efficacy was a noninferiority comparison of mean change in two-hour IOP and zero-hour IOP at the end of treatment compared to the respective baseline IOP. Safety was analyzed by comparing the frequency of the observed adverse events (AEs) between the two groups.

Results

FDC brinzolamide/timolol produced comparable and non-inferior IOP-lowering efficacy to FDC dorzolamide/timolol. The IOP reductions ranged from 6.55 to 8.36 mmHg in FDC brinzolamide/timolol group and from 5.37 to 7.55 mmHg in FDC dorzolamide/timolol group. Fewer subjects in FDC brinzolamide/timolol group experienced ocular AEs as compared with FDC dorzolamide/timolol group (9.9% vs. 26.4%), especially ocular hyperemia (2.7% vs. 22.7%).

Conclusion

FDC of brinzolamide 1%/timolol 0.5% affords an ocular comfort advantage with a clinically meaningful reduction in IOP that was non-inferior to FDC of dorzolamide 2%/timolol 0.5% in Indian patients with POAG and ocular hypertension.

## Introduction

Glaucoma, “the silent thief of sight,” is the leading cause of irreversible blindness in the world [1,2]. Environmental factors, genetics, ethnicity, race, comorbidities, or immune-mediated processes can predispose to glaucoma [2]. However, the central event in all patients with glaucoma is apoptosis of retinal ganglion cells and axons of the optic nerve, subsequently degenerative optic neuropathy, visual field defects, and progressive loss of vision [3].

At the current global prevalence rate of 3.54%, approximately 111.8 million would be glaucomatous worldwide by the end of 2040, with a disproportionate increase predicted in Asia and Africa [4,5]. The prevalence of glaucoma in India is expected to increase drastically in urban and rural areas due to an aging population and increasing comorbidities like diabetes mellitus, hypertension, and cardiovascular disorders [6,7]. Glaucoma Society of India estimates the current prevalence of glaucoma between 2% and 13%, which is likely to affect 12 million Indians, contributing to 12.8% of irreversible blindness [8]. Large population-based studies in India over the previous decade have observed that primary open-angle glaucoma (POAG) is more common than primary angle-closure glaucoma (PACG), contrary to earlier studies [9,10].

Of all the modifiable and non-modifiable risk factors attributable to glaucoma, perhaps the only factor that can be modifiable currently is intraocular pressure (IOP). Maintaining a designated target IOP mitigates any further retinal ganglion cell loss; hence, IOP reduction is of utmost importance to prevent further progression of field defects and vision loss [11-13].

Approximately two-thirds of POAG and ocular hypertension patients, over the course of their disease, need more than one drug or combination therapy to maintain the designated target IOP, due to which adherence to glaucoma medication and compliance to treatment is a major challenge [14-16].

In this regard, fixed-dose combinations (FDCs) have greater potential in the initial management of glaucoma by reducing the adverse effects by minimizing the exposure to preservatives, decreasing chances of washout of the drugs, reducing the frequency of medication leading to improved compliance, reducing treatment cost and improved treatment efficacy due to their complementary action [17,18].

Treatment options for glaucoma include pharmacotherapy, laser therapy, and surgical management. However, pharmacotherapy constitutes the primary treatment option in the initial management of POAG. A wide array of anti-glaucoma drugs based on different mechanisms of action are available currently, like alpha agonists, beta-blockers (timolol), carbonic anhydrase inhibitors (CAIs) (brinzolamide, dorzolamide), prostaglandin analogs, cholinergic agents, and osmotic agents [19].

Brinzolamide and timolol cause a net reduction in the IOP by decreasing the production of aqueous humor by inhibiting the enzyme carbonic anhydrase and blocking the beta receptors on the ciliary epithelium, respectively. This forms the basis for using it as a FDC due to its complementary action [19,20].

Earlier published studies on IOP reduction with the combination of brinzolamide and timolol displayed a superior reduction in IOP to either brinzolamide or timolol alone due to their complementary actions [20]. Efficacy studies of FDC of brinzolamide and timolol have yielded 30-33% IOP reduction from the untreated baseline IOP of 25-27 mmHg in patients with POAG and ocular hypertension [11]. On comparing the FDC brinzolamide/timolol vs. FDC dorzolamide/timolol, the IOP lowering effect was statistically significant, clinically relevant, and non-inferior in the former than the latter [21]. FDC of brinzolamide/timolol was also found to have better ocular tolerability and ocular comfort than that of dorzolamide/timolol in prior studies [21,22]. In the Silver et al. study, a lesser proportion of patients had ocular discomfort with brinzolamide 1%, and a lower ocular discomfort score of 1.3 units was observed when compared with dorzolamide 2%. The greater ocular comfort afforded by brinzolamide could be due to its near physiologic pH formulation of 7.2, compared to dorzolamide, which is formulated at an acidic pH of 5.6 [18,22,23]. FDC of brinzolamide/timolol is approved and available for the treatment of POAG and ocular hypertension in the Western population. However, data on the Indian population are currently lacking.

Hence, in our study, we aimed to compare the efficacy and safety of twice-daily FDC brinzolamide 1%/timolol 0.5% ophthalmic suspension with that of twice-daily FDC dorzolamide 2%/timolol 0.5% ophthalmic solution on IOP reduction in Indian patients with POAG and ocular hypertension.

## Materials and methods

This was a 12-week randomized, phase 3, open-label, comparative study conducted in India at nine sites between November 2014 and June 2015. The study was registered with Clinical Trial Registry India (Registration Number CTRI/2014/09/005062, dated 25/09/2014), approved by the respective institutional ethical committees, and conducted with strict adherence to the tenets of the Declaration of Helsinki.

After obtaining informed and written consent, subjects were screened for eligibility. Subjects aged between 18 and 75 years of either gender, newly diagnosed or known POAG with IOP not adequately controlled on monotherapy of either CAIs, beta-blockers, or any other IOP-lowering agent, with an IOP range of 24-36 mmHg, were included in the study.

Subjects with other forms of glaucoma, history of hypersensitivity to oral or topical CAIs, beta-blockers, and sulfonamide drugs, or any of the following ophthalmic conditions (acute or chronic conjunctivitis, dry eye syndrome, diabetic retinopathy, history of intraocular or laser surgeries in the preceding three months) were excluded from the study.

The key exclusion criteria were pregnant or lactating women, women of childbearing potential who were not using contraceptives, uncontrolled hypertension, severe, unstable, or uncontrolled cardiovascular or pulmonary disease, concurrent systemic steroidal medication, a history of hematologic disorders other than mild anemia, non-compliance or unwillingness to adhere to the study.

Enrolled subjects were randomized into two cohorts: group A received FDC brinzolamide 10 mg/mL/timolol 5mg/mL ophthalmic suspension (Brinzotim®, Sun Pharma, Mumbai, India), and group B received FDC dorzolamide 20 mg/mL/timolol 5mg/mL ophthalmic solution. Block randomization (block size of four) was implemented, and a random allocation sequence was generated with all the necessary precautions to avoid bias. A list of random numbers was provided. The sponsor’s team generated the random allocation sequence, the site investigator enrolled the subjects, and the site investigator/site personnel assigned them to the intervention as per the randomization scheme. There was no blinding since this was an open-label study.

One drop of assigned FDC was administered twice daily in the affected eye(s) for 12 weeks. IOP was noted at zero and two hours at all visits, including baseline and at weeks 4, 8, and 12, using an applanation tonometer. The primary efficacy analysis included a change in zero-hour IOP and a change in two-hour IOP at the end of treatment compared to the baseline visit. The secondary efficacy analysis included evaluation of global impression by both patients and investigators (not assessed, very much improved, much improved, minimally improved, no change, minimally worse, much worse, very much worse). Safety analysis was based on the frequency of treatment-emergent adverse events (AE). No changes were made to the study methods or outcomes after the commencement of the trial.

Statistical methods

The sample size of 221 subjects was found to be sufficient to demonstrate non-inferiority of the test product (group A) compared to the reference product (group B) for the primary efficacy parameter assuming a non-inferiority margin of 1.5 mm of Hg, and alpha error 0.025, and power 0.85.

For the primary efficacy endpoint, statistical analysis was done using a paired t-test (within groups), an unpaired t-test, and an analysis of covariance (ANCOVA) (between groups); 95% CIs and p-values were provided. p < 0.05 was considered as the level of significance. Missing observations were treated using the last observation carried forward (LOCF) method. For the secondary efficacy analysis, the p-value was calculated using a two-tailed chi-square test for all assessment variables except “not assessed.” Safety was assessed by evaluating AEs at each visit and summarizing them using frequencies and relative frequencies, categorized by preferred term and system organ class, which was tabulated according to severity and relationship to the study drug. Statistical analysis was performed using SAS software version 9.2 (SAS Institute Inc., Cary, NC) [24].

## Results

**Figure 1:**
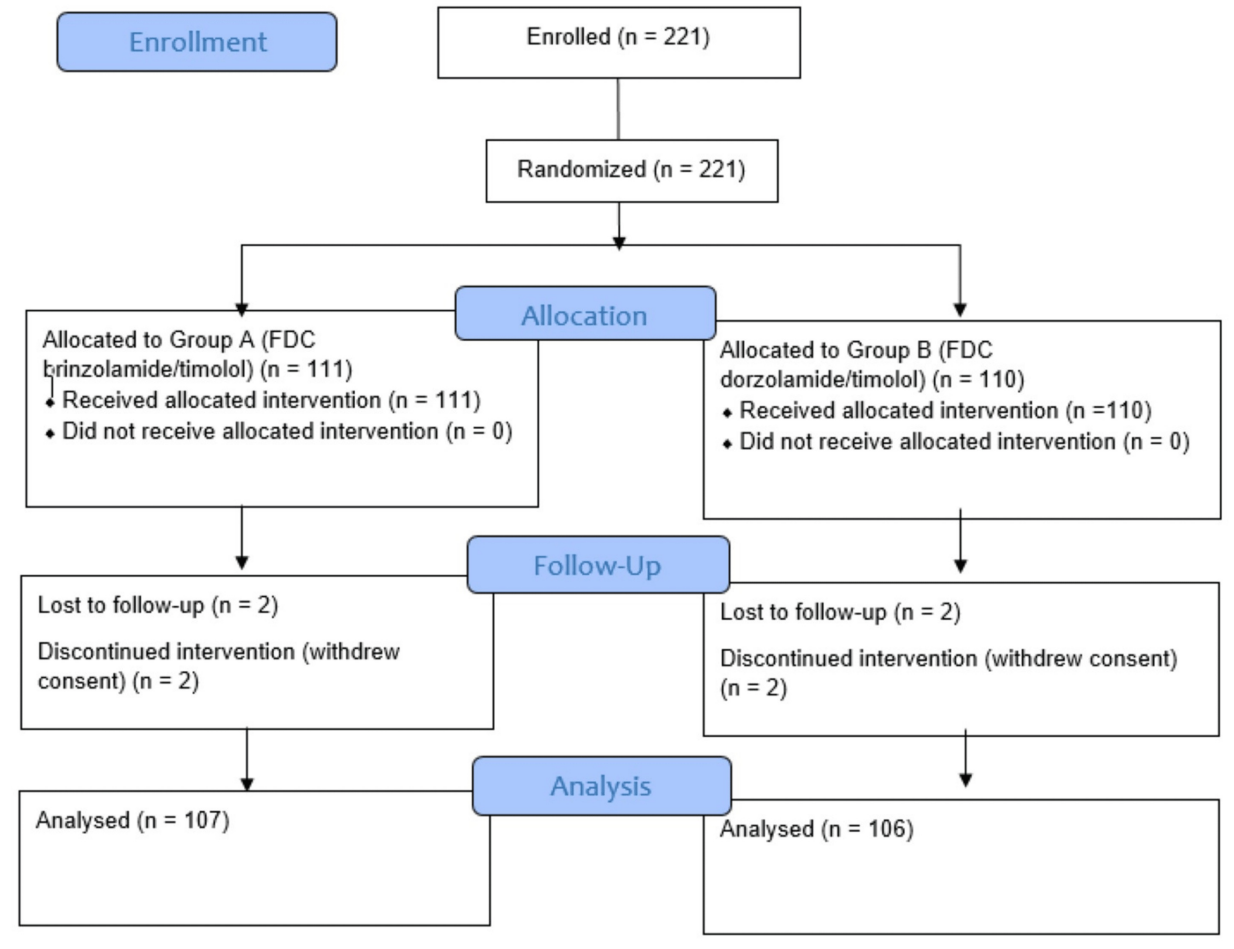
CONSORT CONSORT 2010 statement depicting the enrollment and randomization of study subjects into group A and group B CONSORT, Consolidated Standards of Reporting Trials; FDC, fixed-dose combination

A total of 221 subjects (111 subjects in group A brinzolamide/timolol and 110 subjects in group B dorzolamide/timolol) were enrolled and randomized into the study and received at least one dose of the study drug and have at least one post-treatment assessment. Both groups were comparable, and there was no clinical or statistically significant difference observed in the vital signs or laboratory parameters among both groups. Results are presented for the intent-to-treat (ITT) population (Table 1 and Table 2) along with the Consolidated Standards of Reporting Trials (CONSORT) 2010 statement presented in Figure 1.

**Table 1:** Demographic data IOP, intraocular pressure; FDC, fixed-dose combination

Parameter	Group A FDC brinzolamide/timolol (n = 111)	Group B FDC dorzolamide/timolol (n = 110)
Age, years (mean ± SD)	56.3 ± 13.56	55.9 ± 13.10
Male, n (%)	60 (54.1)	60 (54.5)
Female, n (%)	51 (45.9)	50 (45.5)
Baseline IOP (zero hours), mmHg (mean ± SD)	27.12 ± 3.13	27.04 ± 2.77
Baseline IOP (two hours), mmHg (mean ± SD)	22.40 ± 3.74	22.16 ± 3.90

**Table 2:** Subject disposition FDC, fixed-dose combination

Parameter	Group A FDC brinzolamide/timolol, n (%)	Group B FDC dorzolamide/timolol, n (%)
Enrolled subjects	111 (100.0)	110 (100.0)
Completed the study	107 (96.4)	106 (96.4)
Discontinued the study	4 (3.6)	4 (3.6)
Consent withdrawn	2 (1.8)	2 (1.8)
Lost to follow-up	2 (1.8)	2 (1.8)

Reduction in mean zero-hour IOP

In group A (brinzolamide/timolol), the mean IOP at zero hours prior to the instillation of the drug at baseline (day 0) visit was 27.12 ± 3.13 mmHg, and at day 84 visit was 18.84 ± 3.69 mmHg. This mean change (reduction) of 8.36 mmHg in IOP at the end of 12 weeks was statistically significant (p < 0.05) within the group (Table 3).

**Table 3:** Reduction of mean zero-hour IOP in group A Treatment effect on zero-hour IOP prior to instillation of the drug in group A FDC brinzolamide/timolol. *Statistically significant difference from baseline (p < 0.05). FDC, fixed-dose combination; IOP, intraocular pressure

Group A	Day 0	Day 28	Day 56	Day 84
No. of subjects	(n = 111)	(n = 111)	(n = 110)	(n = 107)
Mean ± SD	27.12 ± 3.13	23.03 ± 3.63	20.90 ± 3.86	18.84 ± 3.69
95% CI of mean	26.54, 27.71	22.35, 23.71	20.17, 21.63	18.14, 19.55
Mean change	-	-4.09*	-6.25*	-8.36*

In group B (dorzolamide/timolol), the mean IOP at zero hours prior to the instillation of the drug at baseline (day 0) visit was 27.04 ± 2.77 mmHg, and at day 84 visit was 19.59 ± 4.67 mmHg. This mean change (reduction) of 7.55 mmHg in IOP at the end of 12 weeks was statistically significant (p < 0.05) within the group (Table 4).

**Table 4:** Reduction in mean zero-hour IOP in group B Treatment effect on zero-hour IOP prior to instillation of the drug in group B FDC dorzolamide/timolol. *Statistically significant difference from baseline (p < 0.05). FDC, fixed-dose combination; IOP, intraocular pressure

Group B	Day 0	Day 28	Day 56	Day 84
No. of subjects	(n = 110)	(n = 110)	(n = 110)	(n = 106)
Mean ± SD	27.04 ± 2.77	22.61 ± 4.15	21.04 ± 4.35	19.59 ± 4.67
95% CI of mean	26.52, 27.56	21.83, 23.40	20.22, 21.86	18.69, 20.49
Mean change	-	-4.43*	-6.00*	-7.55*

A statistically significant difference in the mean zero-hour IOP from baseline was observed in group A on all follow-up visits. However, the observed mean change (reduction) in zero-hour IOP in group A (brinzolamide/timolol) was non-inferior and statistically insignificant compared to group B (dorzolamide/timolol) at all visits (p > 0.05).

Reduction in mean two-hour IOP

In group A (brinzolamide/timolol), the mean IOP two hours after the instillation of the drug at baseline (day 0) visit was 22.40 ± 3.74 mmHg, and at day 84 visit was 16.01 ± 3.52 mmHg. This mean change (reduction) of 6.55 mmHg in IOP at the end of 12 weeks was statistically significant (p < 0.05) within the group (Table 5).

**Table 5:** Reduction in mean two-hour IOP in group A Treatment effect on two-hour IOP after instillation of the drug in group A FDC brinzolamide/timolol. *Statistically significant difference from baseline (p < 0.05). FDC, fixed-dose combination; IOP, intraocular pressure

Group A	Day 0	Day 28	Day 56	Day 84
No. of subjects	(n = 111)	(n = 111)	(n = 110)	(n = 107)
Mean ± SD	22.40 ± 3.74	19.61 ± 3.77	17.93 ± 3.47	16.01 ± 3.52
95% CI of mean	21.70, 23.11	18.90, 20.32	17.28, 18.59	15.33, 16.68
Mean change	-	-2.79*	-4.49*	-6.55*

In group B (dorzolamide/timolol), the mean IOP two hours after the instillation of the drug at baseline (day 0) visit was 22.16 ± 3.90 mmHg, and at day 84 visit was 16.83 ± 4.12 mmHg. This mean change (reduction) of 5.37 mmHg in IOP at the end of 12 weeks was statistically significant (p < 0.05) within the group (Table 6).

**Table 6:** Reduction in mean two-hour IOP in group B Treatment effect on two-hour IOP after instillation of the drug in group B FDC dorzolamide/timolol. *Statistically significant difference from baseline (p < 0.05). FDC, fixed-dose combination; IOP, intraocular pressure

Group B	Day 0	Day 28	Day 56	Day 84
No. of subjects	n = 110	n = 110	n = 110	n = 106
Mean ± SD	22.16 ± 3.90	19.01 ± 4.20	17.87 ± 3.93	16.83 ± 4.12
95% CI of mean	21.42, 22.89	18.22, 19.81	17.13, 18.62	16.04, 17.62
Mean change	-	-3.14*	-4.28*	-5.37*

A statistically significant difference in mean two-hour IOP was observed in group A at all follow-up visits. However, the observed mean change (reduction) in two-hour IOP in group A (brinzolamide/timolol) was non-inferior and statistically insignificant compared to group B (dorzolamide/timolol) at all visits (p > 0.05).

Global impression by patients and investigators

**Figure 2:**
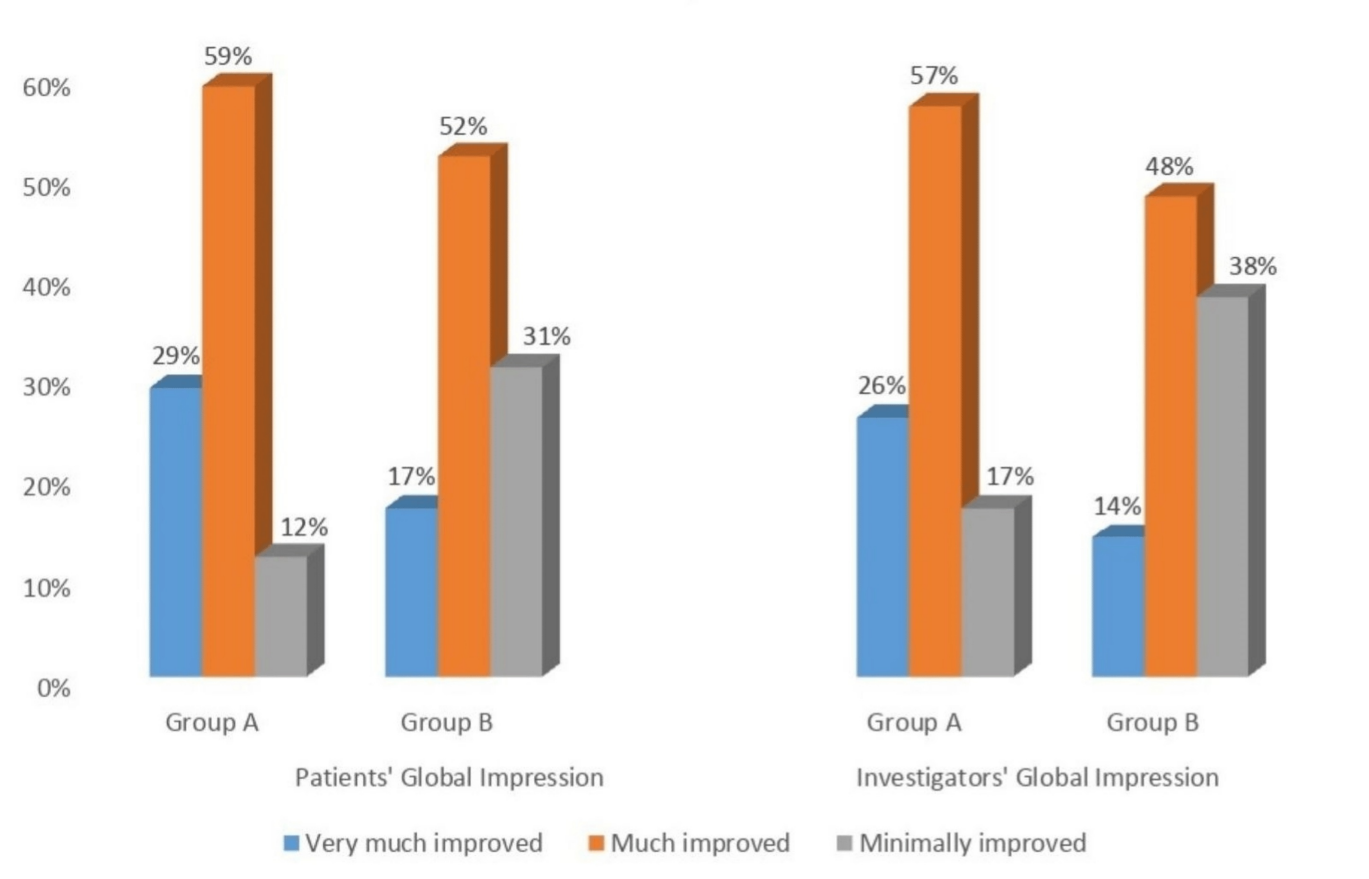
Global impressions by patients and investigators Subjective impression of patients and investigators toward treatment in group A (FDC brinzolamide/timolol) and group B (FDC dorzolamide/timolol) FDC, fixed-dose combination

About four-fifths of the patients and investigators in group A (brinzolamide/timolol) and about two-thirds of the patients and investigators in group B (dorzolamide/timolol) evaluated their condition to have either very much improved or much improved, following usage of the medication (Figure 2). There was no significant difference between the two groups.

Safety

An overview of AEs is presented in Table 7. Twelve subjects (10.8%) in group A (brinzolamide/timolol) and 32 subjects (29.1%) in group B (dorzolamide/timolol) reported at least one AE during the study period. The most common AE observed in brinzolamide/timolol was eye pruritis; however, ocular hyperemia was most frequently observed in the dorzolamide/timolol group. All AEs were mild in severity and not related to the study drug.

**Table 7:** Incidence of adverse events Comparison of incidence of adverse events observed in group A and group B.

Adverse events	Group A FDC brinzolamide/timolol (n = 111), n (%)	Group B FDC dorzolamide/timolol (n = 110), n (%)
Eye disorders	11 (9.9)	29 (26.4)
Excessive eye blinking	2 (1.8)	6 (5.5)
Eye irritation	2 (1.8)	3 (2.7)
Eye pruritis	6 (5.4)	2 (1.8)
Ocular hyperemia	3 (2.7)	25 (22.7)
Blurred vision	0	1 (0.9)
General disorders	1 (0.9)	0
Pyrexia	1 (0.9)	0
Nervous system disorders	0	3 (2.7)
Headache	0	3 (2.7)

## Discussion

This study demonstrates that twice daily FDC brinzolamide 1%/timolol 0.5% ophthalmic suspension provides clinically relevant IOP reductions in subjects with POAG and/or ocular hypertension in India. Mean IOP reduction with FDC brinzolamide/timolol at the end of 12 weeks ranged from 6.55 to 8.36 mmHg, representing 29% to 31% reductions from baseline, compared to FDC dorzolamide/timolol, wherein the mean IOP reduction from the baseline was 5.37 to 7.55 mmHg (24% to 28%). The IOP reduction observed with FDC brinzolamide/timolol was comparable and non-inferior to FDC dorzolamide/timolol at both zero and two hourly assessments.

A similar study in the Western population, comparing the FDC of brinzolamide/timolol vs. FDC of dorzolamide/timolol, by Manni et al. among 437 patients observed differences in mean IOP to be numerically favoring the former in nine out of the 12 study visits and range of mean IOP reductions from 7.2 to 9.2 mmHg in the brinzolamide/timolol group compared to 7.4 to 8.9 mmHg for dorzolamide/timolol, establishing non-inferiority of brinzolamide/timolol compared to dorzolamide/timolol in reducing the IOP from the baseline, similar to our observations [21]. Further, a similar study by Michaud et al. also inferred a non-inferior IOP reduction in the brinzolamide/timolol (-14.2% to -21.9%) treated subjects compared to dorzolamide/timolol (-14.1% to -21.2%) group [25]. 

The complementary IOP lowering effect of the FDC brinzolamide/timolol observed in our study, with a 29% to 31% reduction in IOP at the end of 12 weeks, is especially useful among POAG and/or ocular hypertensives not controlled by monotherapy alone [11,20,25]. Similar findings of complementary IOP reduction with FDC brinzolamide/timolol were noted in the Kaback et al. study, wherein the reduction in IOP at the end of three months was much more profound in those patients who were put on brinzolamide/timolol (-14.0%) than those on timolol alone (-4.4%) [20,25]. A similar study by Yoshikawa et al. also opined a greater complementary IOP mean reductions from baseline with FDC brinzolamide/timolol compared to timolol alone after eight weeks of therapy [26].

However, contrasting results compared to that of our analysis were noted in the Galose et al. study, who opined that IOP reduction by FDC of dorzolamide/timolol was more compared to that of FDC of brinzolamide/timolol [18]. This discrepancy could be due to a small sample size of 73 subjects and a statistically significant higher baseline IOP in the dorzolamide/timolol group (29.53 ± 5.99 mmHg) compared to that of the brinzolamide/timolol group (24.14 ± 4.49 mmHg) in their study.

The global impression of treatment was better among both patients and investigators in group A (brinzolamide/timolol) than that of group B (dorzolamide/timolol). This, perhaps, may have a correlation with fewer subjects in group A experiencing ocular AEs as compared with group B (9.9% vs. 26.4%), especially ocular hyperemia (2.7% vs. 22.7%). Observations of fewer incidences of ocular hyperemia in the FDC brinzolamide/timolol group compared to dorzolamide/timolol were also discerned in the Mundorf et al. study, wherein the mean discomfort scores (1.4 ± 1.6 vs. 2.9 ± 2.5, respectively; p < 0.0001) and patients drug preference (1.5 ± 1.5 vs. 3.3 ± 2.5, respectively; p < 0.0001), both parameters point toward a favorable response to FDC brinzolamide/timolol [27]. Observations similar to ours were also noted in studies by Stewart et al. and Barnebey et al., wherein dorzolamide-treated patients experienced a higher degree of ocular pain following drug instillation compared to brinzolamide and a switch to brinzolamide improved patient comfort [28,29]. This increased ocular AEs observed in the FDC of the dorzolamide/timolol treated group was due to the solution being formulated at a pH of 5.6. Our safety data are consistent with that of studies published by Manni et al. and Michaud et al. [21,25]. The reduced ocular AEs observed with FDC brinzolamide/timolol were due to the fact that it was formulated at close to a physiologic pH of 7.2 [18,27].

Limitations

The study included subjective parameters of assessment, including subjective scoring of global impression scales by both patients and investigators. Since the study design was open-label, there is a likelihood of risk of bias in scoring. There were also increased chances of missing data due to knowledge of treatment. However, care was taken to ensure that there was negligible missing data, and it was approximately equal for both groups. Apart from baseline and follow-up visits, drops had to be administered twice daily by patients themselves. Hence, there is a risk of non-compliance to therapy.

## Conclusions

In this study on Indian patients with POAG and ocular hypertension, FDC of brinzolamide 1%/timolol 0.5% ophthalmic suspension administered twice daily produced a clinically meaningful reduction in IOP over a period of 12 weeks. However, the reduction in IOP was non-inferior to that seen with FDC of dorzolamide 2%/timolol 0.5% ophthalmic solution. Also, there was no difference in global impression by both patients and investigators between the two groups. Despite similar efficacy, fewer ocular AEs, including ocular hyperemia, were reported with the FDC of brinzolamide 1%/timolol 0.5% compared to the FDC of dorzolamide 2%/timolol 0.5%. Therefore, FDC of brinzolamide 1%/timolol 0.5% will encourage compliance to treatment in real-world clinical practice, which is desirable in the management of glaucoma in whom the IOP reduction is suboptimal with monotherapy alone.
